# Relationship between histological mixed-type early gastric cancer and lymph node metastasis: A systematic review and meta-analysis

**DOI:** 10.1371/journal.pone.0266952

**Published:** 2022-04-15

**Authors:** Shufan Yang, Xin Gu, Rui Tao, Jiahui Huo, Zhen Hu, Fei Sun, Jinbin Ni, Xiaoyun Wang

**Affiliations:** 1 Nantong University, Nantong City, Jiangsu, P.R. China; 2 The Affiliated Wuxi No.2 People’s Hospital of Nanjing Medical University, Wuxi City, Jiangsu, P.R. China; 3 The Affiliated Wuxi Clinical College of Nantong University, Wuxi City, Jiangsu, P.R. China; Texas Tech University Health Science, Lubbock, UNITED STATES

## Abstract

The clinicopathological features of early gastric cancer (EGC) with mixed-type histology (differentiated and undifferentiated) are incompletely understood, and the capacity of endoscopic submucosal dissection (ESD) to treat mixed-type cancer remains controversial. This systematic review analyzed the rate of lymph node metastasis (LNM) in mixed-type EGC. We gathered articles published up to February 21, 2021, that analyzed the relationship between LNM and mixed-type EGC from Embase, PubMed, and Web of Science. The primary outcome was the LNM rate associated with different histological types of EGC, and the secondary outcomes were the odds ratios (ORs) for LNM risk factors among EGC patients. From the 24 studies included in this meta-analysis, the overall rate of LNM in predominantly differentiated mixed-type (MD) EGC was 12%, whereas the LNM rate in predominantly undifferentiated mixed-type (MU) EGC was 22%. We further divided these studies into 2 groups according to the depth of invasion. In mixed-type mucosal EGC, the pooled LNM rate was 15%; in submucosal EGC, the rate was 33% for MU, which was higher than the rates for pure types (pure differentiated type, 13%; pure undifferentiated type, 21%; p<0.05). The LNM rate of MD was 20%, it was higher than those of the pure differentiated type and nearly the same as pure undifferentiated type. Other pooled statistics showed that submucosal invasion, pure undifferentiated EGC, and mixed-type EGC were independent risk factors for LNM. This meta-analysis showed that MD submucosal EGC has a high rate of LNM and is highly correlated with LNM; thus, the management of MD EGC as purely differentiated EGC according to the indications for ESD is inappropriate, and the mixed type should be added as a parameter in these indications.

## 1. Introduction

Gastric cancer is the fifth most common cancer and the third leading cause of cancer-related deaths worldwide [1]. The prevalence of early gastric cancer (EGC) is higher in East Asian countries, such as China, Japan, and Korea, than in western countries [2, 3], and nearly half of all global cases in 2017 occurred in China [4]. These East Asian countries have made efforts to overcome gastric cancer in the past several decades. For example, Korea and Japan have developed gastric cancer screening programs to reduce morbidity, resulting in the detection of gastric cancers at early stages [5, 6]. Endoscopic treatments, especially endoscopic submucosal dissection (ESD), have been adopted as the main treatment options for EGC in Japan [7], as they contribute to high 5-year overall survival rates and improve patient quality of life [8]. Some researchers, therefore, believe that China should adopt a similar strategy to further reduce the burden of stomach cancer [9].

The EGC indications for ESD published by the Japanese Gastric Cancer Association [10] generally divide gastric cancer into differentiated and undifferentiated types. No separate indication exists for mixed-type gastric cancer [11]; instead, a mixed-type specimen with predominantly differentiated components is classified as differentiated, whereas one with predominantly undifferentiated components is deemed undifferentiated. However, due to advances in histopathological biopsy, mixed components are commonly identified in EGC patients during reviews of ESD specimens [12].

A lesion suitable for ESD demonstrates a negligible risk of lymph node metastasis (LNM). Statistics from the Japanese National Cancer Center Hospital and Cancer Institute Hospital [10] show that the rate of LNM in the mucosa is low in both differentiated and undifferentiated gastric cancer patients. Gotoda et al. [13] also demonstrated that submucosal gastric lesions with a depth of invasion <500 μm present a low risk of LNM and that those less than 30 mm in diameter, associated with differentiated gastric cancer or with no lymphovascular invasion showed no risk of LNM. However, the rate of LNM in mixed-type EGC patients is considerably higher [14]. For example, Takizawa et al. [15] reported that the rate of LNM in mixed-type mucosal gastric cancer patients is 19%, much higher than in those with pure types(6%). Other studies have shown the mixed histological type to have aggressive biological behaviors [16, 17]. This high LNM rate has made the curability assessment and subsequent treatment controversial. The prognosis—especially the LNM rate associated with mixed-histological EGC—is still unclear. Our study aimed to determine whether mixed histology affects the rates of curative resection and, especially, LNM and whether there are other contributing factors. Because a single study may lack the power to provide a reliable conclusion, we performed a systematic review to evaluate the risk of LNM in mixed-type EGC patients.

## 2. Methods

Our study followed the PRISMA guidelines for reporting on systematic reviews and meta-analyses (http://www.prisma-statement.org/). We registered the study at the International Prospective Register of Systematic Reviews (https://www.crd.york.ac.uk/PROSPERO/, No. CRD42021240673).

### 2.1 Searching strategy

We conducted a computer-aided search of PubMed/Medline, Embase, and Web of Science for articles that analyzed LNM rates in mixed-type EGC patients published up to Feb 21, 2021. The following keywords were used in the search: “early gastric cancer OR early stomach neoplasms OR intramucosal gastric cancer OR submucosal gastric cancer OR intramucosal gastric carcinoma OR submucosal gastric carcinoma OR mucosal gastric cancer OR mucosal gastric carcinoma OR submucosal invasive gastric cancer” AND “mixed histology OR mixed OR mixed type OR histologic heterogeneity OR histologic mixed type” AND “lymph node metastasis OR LN metastasis OR LNM.” We used both free text and MeSH searches for keywords. The language was not limited.

### 2.2 Inclusion and exclusion criteria

Studies meeting all of the following criteria were included: patients with EGC who underwent surgical resection or were treated with additional surgery after ESD, histology type assessment included mixed (undifferentiated and differentiated) types, study outcome suggested the evaluation of LNM expansion in mixed-type cancer after surgeries, study design of any type, human subjects used, and availability of the full-text publication.

Studies meeting at least one of the following exclusion criteria were excluded from our analysis: review articles, meta-analysis articles, publications with incomplete data, and case reports.

Four authors (Shufan Yang, Jiahui Huo, Rui Tao, and Xin Gu) performed the study selection independently according to the inclusion and exclusion criteria. Titles, abstracts, and full texts were evaluated to select studies for inclusion in the meta-analysis. If discrepancies existed, a fifth researcher (Xiaoyun Wang) made a judgment to ensure accuracy.

### 2.3 Data extraction and quality assessment

The following data were extracted: first author’s last name, publication year, country, study format, total number of cases, number of patients with LNM, patient characteristics (age and sex), and summary of odds ratios (ORs) with 95% confidence intervals (95% CIs) for LNM risk factors considered in the studies, including tumor size, depth of invasion, lymphovascular invasion, presence of ulcers, histological type (mixed and pure undifferentiated). Quality assessment was performed using the Newcastle–Ottawa Scale (NOS) [18].

### 2.4 ESD indications and curability assessment

EGC is a cancer with invasion confined to the mucosa or submucosa, irrespective of the presence of LNM [19]. The International Union Against Cancer TNM guidelines [20] define mucosal (M) cancers as stage pT1a and submucosal (SM) cancers as pT1b. The Japanese Classification [11, 19] of gastric carcinoma submucosal tumors further divides submucosal tumor stages into pT1b1 (submucosal invasion <0.5 mm, SM1) or pT1b2 (submucosal invasion ≥0.5 mm, SM2).

According to the Japanese Gastric Cancer Association guidelines [10], the absolute indication for ESD includes (i) differentiated mucosal cancer without ulceration or (ii) differentiated mucosal cancer ≤3 cm with ulceration. The expanded indication of ESD includes undifferentiated mucosal cancer ≤2 cm without ulceration.

After endoscopic resection, combined with postoperative pathology, an endoscopic curability (eCura) assessment is conducted to classify the resection as eCura A (curative resection), eCura B (expanded curative resection), or eCura C (non-curative resection). eCura A and B classifications require en bloc resection with no lymphovascular invasion and negative resection margins. If the lesion fulfills the absolute indication for ESD, it is considered eCura A; if it fulfills the expanded indication or includes differentiated cancer measuring ≤3 cm with a submucosal invasion depth of SM1, it is considered eCura B. A lesion not meeting the above criteria is considered eCura C. Follow-up with esophagogastroduodenoscopy and an ultrasonography or computed tomography scan to detect metastases is recommended at intervals of 6–12 months for patients with eCura A and B lesions because of their negligible LNM risk [21, 22].

### 2.5 Definition of mixed type

The Japanese Gastric Cancer Classification [11, 19] names the following as common histological types: papillary adenocarcinoma, tubular adenocarcinoma (well-differentiated or moderately differentiated), poorly differentiated adenocarcinoma (solid type or nonsolid type), signet-ring cell carcinoma, and mucinous adenocarcinoma. The World Health Organization (WHO) gastric cancer diagnostic criteria [23, 24] divide the common histological types into papillary adenocarcinoma, tubular adenocarcinoma, poorly cohesive adenocarcinoma (including signet-ring cell carcinoma), and mucinous adenocarcinoma.

Pure differentiated type (PD) gastric cancer includes papillary adenocarcinoma, whereas the well-differentiated or moderately differentiated types include tubular adenocarcinoma; the pure undifferentiated type (PU) includes poorly differentiated adenocarcinoma, mucinous adenocarcinoma, and signet-ring cell carcinoma (poorly cohesive adenocarcinoma); a histological mixed type that consists of both differentiated (papillary adenocarcinoma and well-differentiated or moderately differentiated tubular adenocarcinoma) and undifferentiated components (poorly differentiated adenocarcinoma, mucinous adenocarcinoma, and signet-ring cell carcinoma). The mixed type is further divided into the predominantly differentiated mixed type (MD; differentiated components >50%) and the predominantly undifferentiated mixed type (MU; undifferentiated components >50%) [25].

### 2.6 Data synthesis and statistical analysis

We investigated the correlation between mixed-type EGC and the rate of LNM. We determined a single, pooled rate of LNM for different histological types with 95% CIs. ORs with 95% CIs were used as the primary summary statistic. These statistics were extracted from the studies to estimate the LNM rate and the risk factors for mixed-type EGC. Cochrane’s Q test (chi-square test; χ²) and the I² metric were used to test the heterogeneity of the pooled results. I²<25% indicated no heterogeneity; I² = 25%–50%, moderate heterogeneity; I² = 50%–75%, medium heterogeneity; and I²>75%, extreme heterogeneity. We used a fixed-effects model (the Mantel–Haenszel method) for studies with I²<50% and p<0.05 in this meta-analysis. Otherwise, we used a random-effects model for our analysis. All statistical analysis was performed using the Stata/SE version 12.0 for Windows (StataCorp, College Station, TX, USA).

## 3. Results

### 3.1 Study selection and characteristics

**Fig 1:**
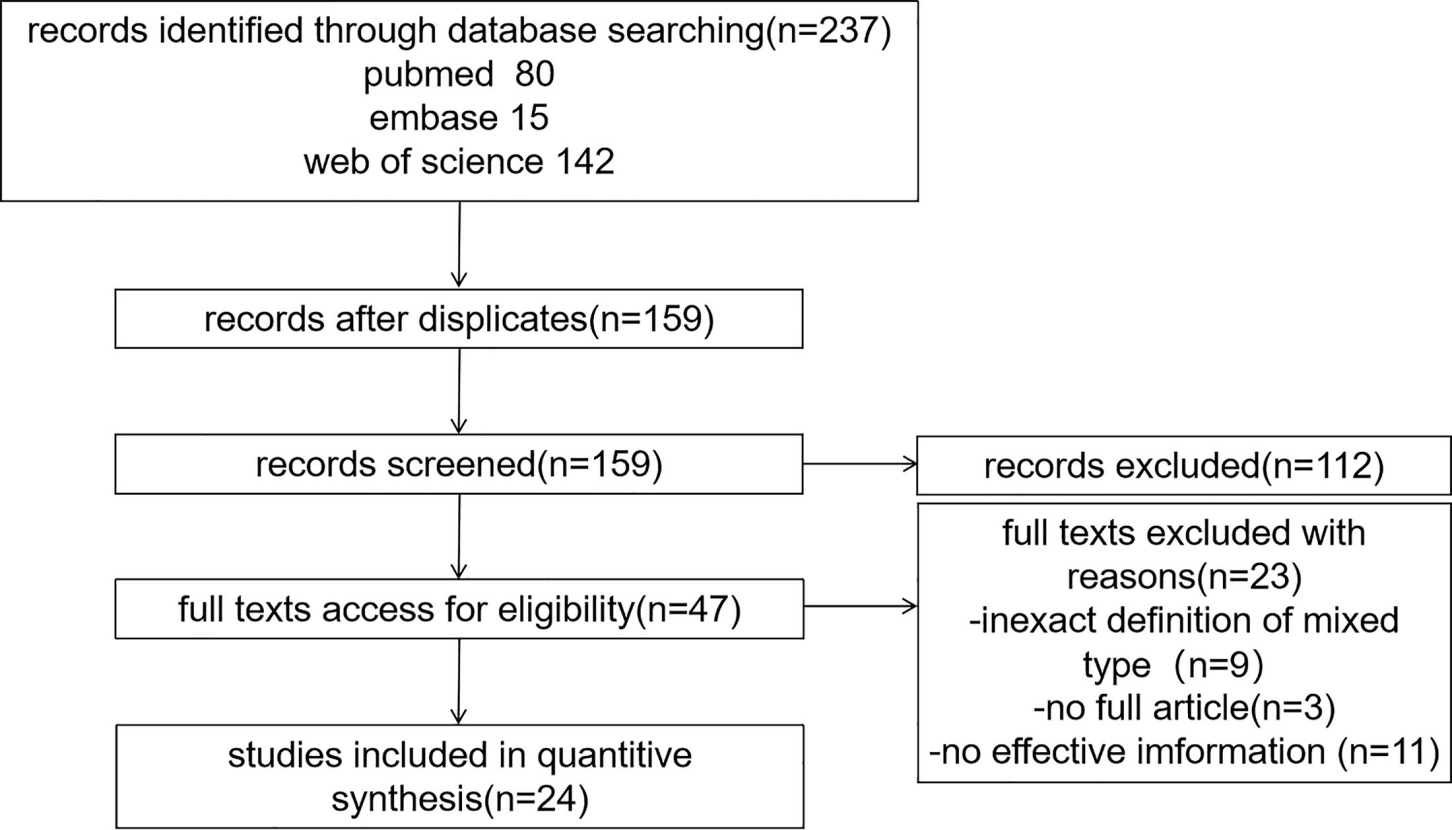
Search results and selection process.

We electronically retrieved 237 unique studies, and 159 studies remained after filtering. After scanning titles and abstracts, 47 citations remained for full-text assessment. The search results and selection process are summarized in Fig 1. Twenty-three studies were excluded for the following reasons: 9 studies did not include histological mixed-type classification [16, 26–33]; 6 did not directly mention the number of LNM cases [34–39]; 3 were considered to have unreliable data [40–42]; 2 were not confined to EGC [12, 43]; and 3 were not accessible online. Finally, 24 studies were deemed eligible for the meta-analysis. Characteristics of the studies and NOS scores included are shown in Table 1.

**Table 1:** Characteristics of included studies.

Author	Publication year	Country	Study format	Total number of cases	Mean age (range/SD)	Sex		Depth of invasion	Mixed type	LNM+/total (N%) in mixed type	NOS assessment scale score
						Male	Female				
Asakawa [62]	2015	Japan	Retrospective	567	59.9±12.3	312	255	m/sm	Mixed type	15/101 (14.9)	6
Chen [53]	2020	China	Retrospective	1596	62 (17–88)	1069	527	m/sm	MD/MU	MD: 22/157 (14.0)	6
										MU: 85/332 (25.6)	
Chen [63]	2017	China	Retrospective	1620	60.9±10.9	1135	485	m/sm	Mixed type	59/201 (29.4)	7
Du [45]	2019	China	Retrospective	621	62.2±10.2	433	188	sm	Mixed type	28/63 (44.4)	6
Hanaoka [25]	2009	Japan	Retrospective	376	N	271	105	sm	MD/MU	MD: 20/104 (19.2)	7
										MU: 23/63 (36.5)	
Horiuchi [50]	2018	Japan	Retrospective	2585	N	N	N	m/sm	MD	10/246 (4.1)	6
Horiuchi [35]	2020	Japan	Retrospective	1425	60 (50–68)	711	714	m/sm	MU	104/525 (19.8)	6
Huh [51]	2013	Korea	Retrospective	2208	N	N	N	m/sm	MU	2/24	6
Ito [52]	2011	Japan	Retrospective	327	N	204	123	m/sm	MD/MU	MD: 9/56 (16.0)	7
										MU: 11/36 (30.6)	
Kim [55]	2020	Korea	Retrospective	2643	N	N	N	m/sm	MD	7/217 (3.2)	8
Kim [44]	2017	Korea	Retrospective	317	N	130	187	sm	MU	44/138 (31.9)	7
Lee [49]	2015	Korea	Retrospective	847	60.2±10.5	615	232	m	MD	11/215 (5.1)	6
Mikami [46]	2018	Japan	Retrospective	279	65.5±9.2	198	81	sm	Mixed type	36/99 (36.4)	6
Miyamae [47]	2016	Japan	Retrospective	239	69 (35–91)	169	70	sm	MD/MU	MD: 14/67 (20.9)	6
										MU: 14/45 (31.1)	
Sekiguchi [56]	2016	Japan	Retrospective	3131	62 (23–88)	2006	1125	m/sm	MD/MU	MD: 115/612 (18.8) MU: 96/469 (20.5)	9
Seo [61]	2019	Korea	Retrospective	1645	59.3±10.9	1084	597	m/sm	Mixed type	26/112 (23.2)	7
Shim [57]	2015	Korea	Retrospective	1039	66.5±9.8	795	244	m/sm	MD	2/28 (7.1)	7
Tajima [48]	2010	Japan	Retrospective	189	64.3±11.4	117	72	sm	Mixed type	32/92 (34.8)	8
Takeuchi [64]	2018	Japan	Retrospective	410	N	257	153	m/sm	Mixed type	5/85 (5.9)	6
Takizawa [15]	2013	Japan	Retrospective	410	61 (29–87)	240	170	m	MD/MU	MD: 6/54 (11.1)	6
										MU: 8/42 (19.0)	
Tang [59]	2020	China	Retrospective	853	N	645	208	m/sm	Mixed type	12/105 (11.4)	7
Yoon [17]	2016	Korea	Retrospective	3419	57.4±11.5	2224	1195	m/sm	MD/MU	MD: 7/54 (13.0)	6
										MU: 25/125 (20.0)	
Zhao [60]	2020	China	Retrospective	302	56.1±12.8	217	85	m/sm	Mixed type	12/37 (32.4)	7
Zhong [58]	2018	China	Retrospective	298	59.5±12.1	206	92	m/sm	Mixed type	12/41 (29.2)	8

NOS, Newcastle–Ottawa Scale; N, not mentioned; m, mucosal cancers; sm, submucosal gastric cancer; MD, predominantly differentiated mixed type; MU, predominantly undifferentiated mixed type.

All included studies were retrospective studies rated with at least 5 stars on the NOS. Six studies reported a depth of invasion confined to the submucosa [25, 44–48]. Outcomes for pT1a gastric cancer were reported in 2 articles [15, 49]. Fourteen studies divided the mixed type into MD and MU for separate analysis [15, 17, 25, 44, 47, 49–57]. ORs with 95% CIs were obtained from all articles, and multivariate analysis was conducted to determine the risk factors of LNM in EGC (Table 2). We obtained ORs with 95% CIs for risk factors for LNM directly from 6 studies [53, 56, 58–61].

**Table 2:** Summary of odds ratios with 95% CIs in included studies as risk factors for lymph node metastasis (results of multivariate analysis).

Study	Tumor size (vs. <2 cm)	Submucosal invasion (vs. intramucosal invasion)	Mixed type (vs. PD)	LVI (vs. absent)	Ulcer (vs. absent)	PU (vs. PD)
Zhong [58]	N	4.58 (1.23–16.97)	5.84 (1.05–32.61)	N	N	4.97 (1.21–30.29)
Sekiguchi [56]	>2, ≤3 cm: 1.7 (1.2–2.5)	SM2: 3.1 (2.2–4.4)	MD: 2.1 (1.5–2.9)	6.7 (5–8.9)	1.7 (1.3–2.2)	1.6 (1.1–2.3)
	>3 cm: 3 (2.2–4.2)		MU: 2.8 (1.9–4.1)			
Tang [59]	>2 cm: 3.59 (1.93–6.69)	1.86 (1.04–3.34)	3.55 (1.5–8.39)	7.86 (3.78–16.33)	2.83 (1.44–5.55)	2.77 (1.4–5.47)
Zhao [60]	>2 cm: 2.153 (1.113–4.164)	3.881 (1.832–8.222)	3.635 (1.272–10.39)	8.797 (2.643–29.277)	N	3.146 (1.352–7.32)
Seo [61]	>2 cm: 1.308 (1.194–1.432)	2.565 (2.168–5.861)	2.360 (1.282–4.343)	9.755 (6.553–14.523)	N	1.657 (1.100–2.495)
Chen [53]	N	N	2.945 (2.039–4.253)	N	N	N

N, not mentioned; SM2, submucosal invasion ≥0.5 mm; PD, pure differentiated type; MD, predominantly differentiated mixed type; MU, predominantly undifferentiated mixed type; PU, pure undifferentiated type; LVI, lymphovascular invasion.

### 3.2 Primary outcomes

#### 3.2.1 Rate of LNM in mixed-type EGC patients

**Fig 2:**
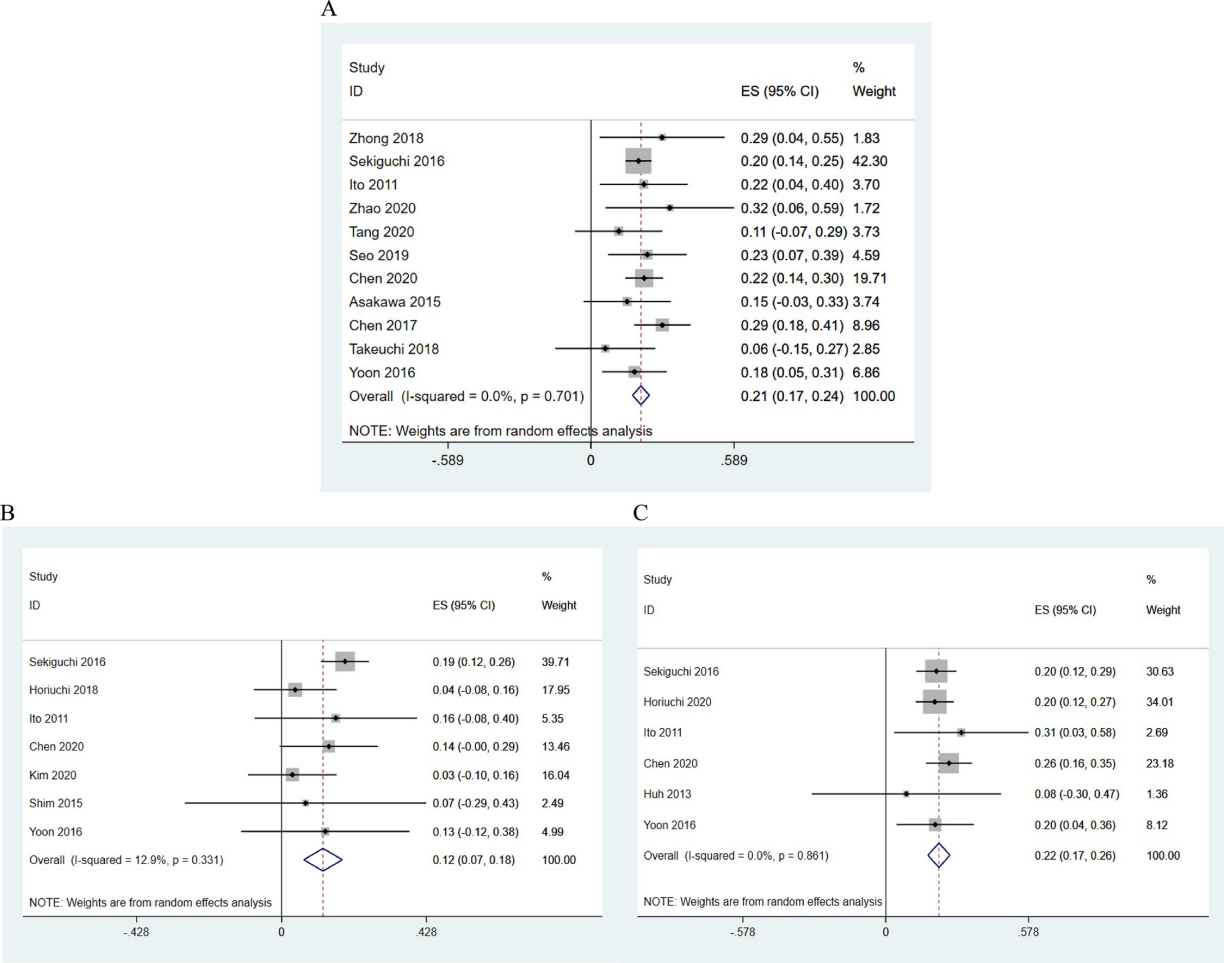
The rate of LNM in mixed-type early gastric cancer (A) overall, (B) for the predominantly differentiated mixed type (MD), and (C) for the predominantly undifferentiated mixed type (MU).

The overall rate of LNM in mixed-type EGC (Fig 2A) was 0.21 (21%; 95% CI: 0.17–0.24, z = 11.57, p = 0.000); no significant heterogeneity was observed (I² = 0.0%, p = 0.701).

Fig 2B presents the LNM rate in MD EGC patients. The pooled rate was 0.12 (12%; 95% CI: 0.07–0.18, z = 4.23, p = 0.000; I² = 12.9%, p = 0.331). The pooled rate of LNM in MU EGC patients was 0.22 (22%; 95% CI: 0.17–0.26, z = 9.43, p = 0.000, I² = 0.0%, p = 0.861), as shown in Fig 2C.

Using fixed-effects models, the pooled results of the LNM statistics from 6 studies indicated that both MD and MU EGC present a high risk of LNM.

#### 3.2.2 Rate of LNM in mucosa and submucosa

**Fig 3:**
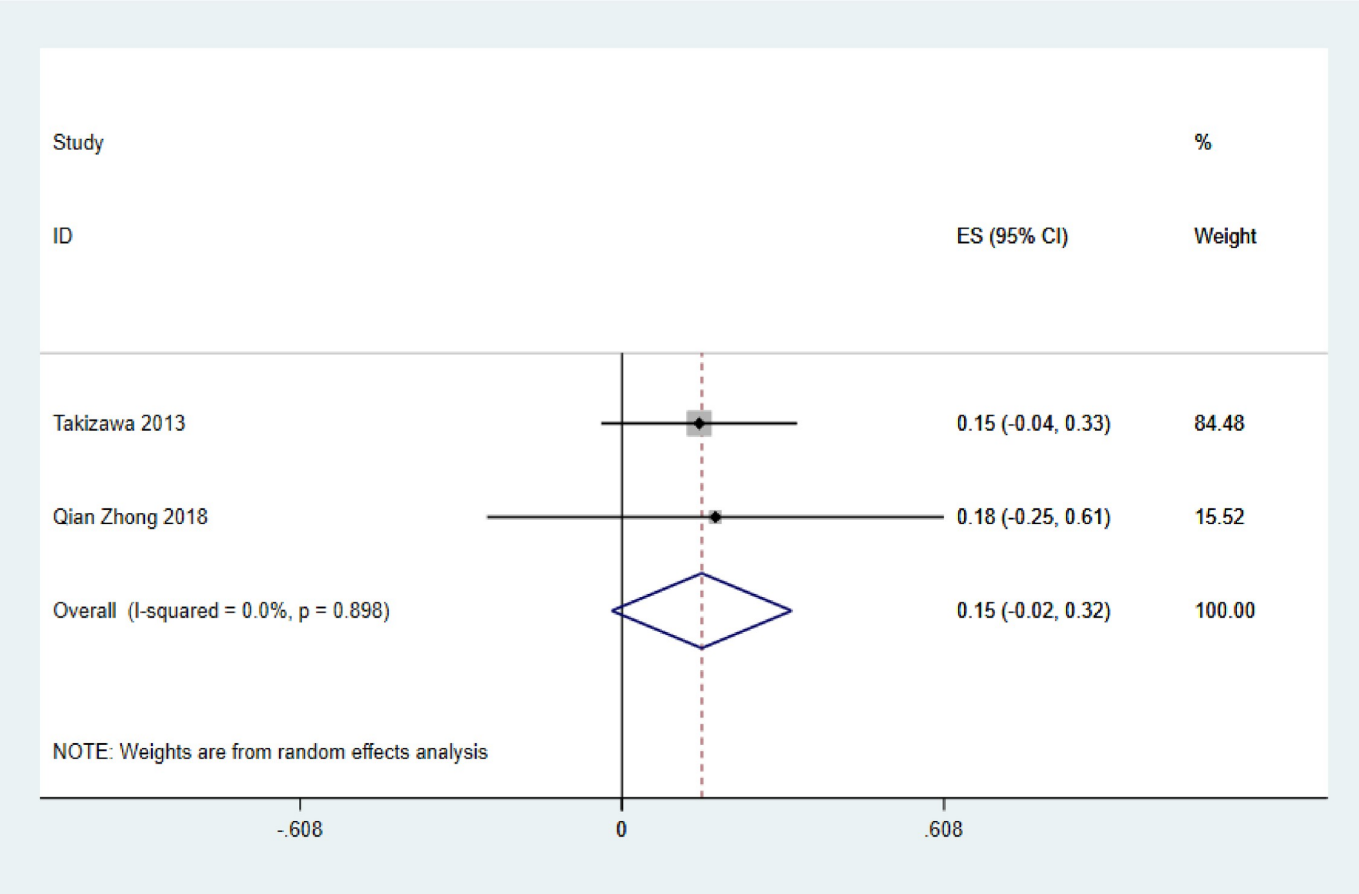
The rate of LNM in mixed-type intramucosal gastric cancer.

In the intramucosal carcinoma studies that reported low rates of LNM, only 2 studies included information on the mixed type (Fig 3). The pooled rate for these was 0.15 (15%; 95% CI: −0.02 to 0.32; z = 1.74, p = 0.082, I² = 0.0%, p = 0.898) and was independent of the overall LNM rate, though the difference was not statistically significant (p>0.05). Of the 2 studies that subdivided statistics into MD and MU, which may show features of metastasis, Takizawa et al. [15] found an LNM rate of 11% in MD and 19% in MU, and Lee et al. [49] presented a rate of 5% in MD.

**Fig 4:**
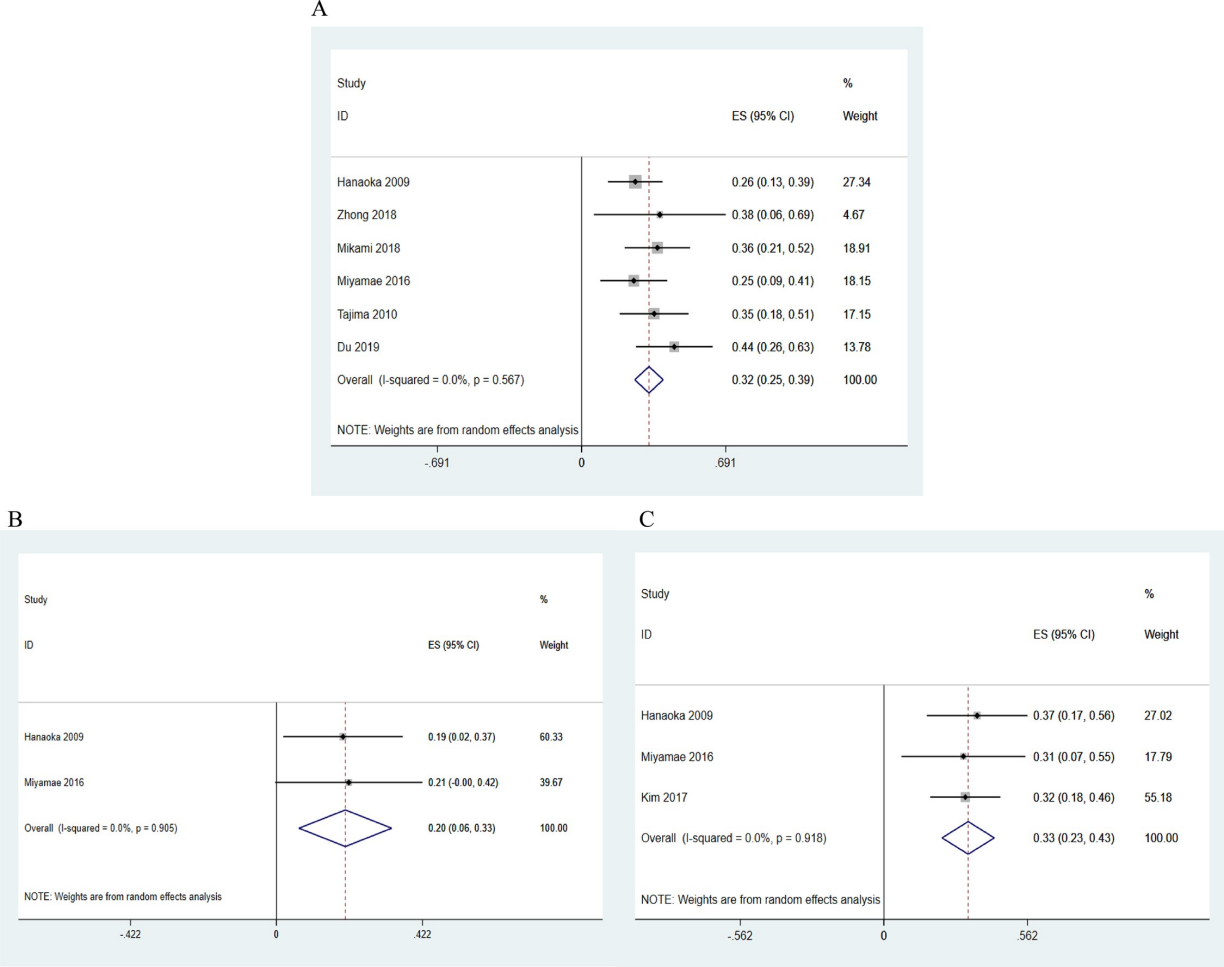
The rate of LNM in submucosal gastric cancer patients: (A) mixed type, (B) predominantly differentiated mixed type (MD), and (C) predominantly undifferentiated mixed type (MU).

A summary of the LNM rate in submucosal gastric cancer patients is presented in Fig 4A. The rate was high (32%, 95% CI: 0.25–0.39, z = 9.26, p = 0.000) with no heterogeneity (I² = 0%, p = 0.567).

Some of these studies also determined histological differences in the results. The rates were 0.20 (20%) for MD (95% CI: 0.06–0.33, z = 2.91, p = 0.004; I² = 0%, p = 0.905; Fig 4B) and 0.33 (33%) for MU (95% CI: 0.23–0.43, z = 6.32, p = 0.000; I² = 0%, p = 0.918; Fig 4C). We further pooled the prevalence of LNM in PU (0.21; 21%) and PD (0.13; 13%) for reference (S1 and S2 Figs).

#### 3.2.3 Risk factors for LNM in ECG

**Fig 5:**
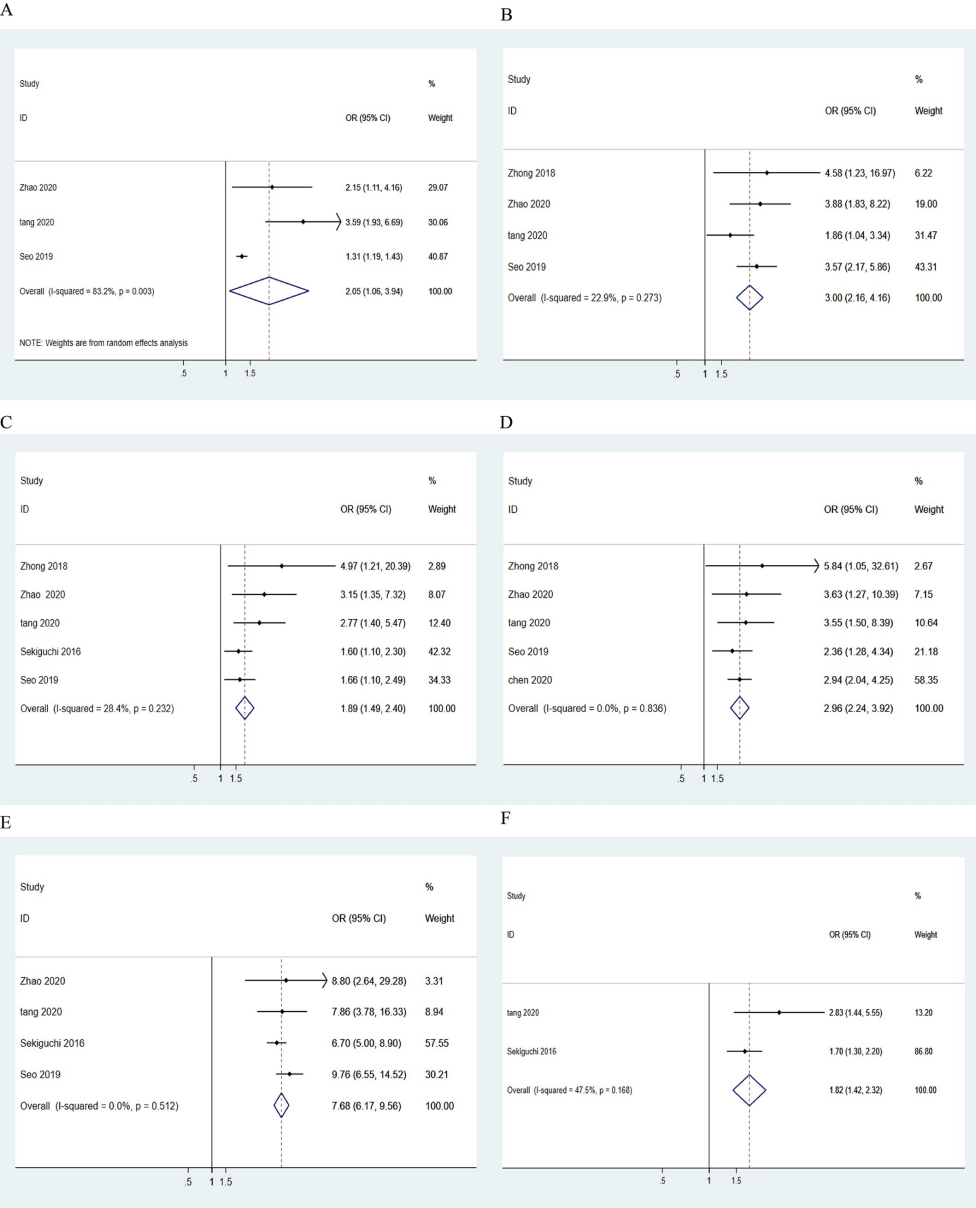
Pooled ORs of (A) tumor size, (B) depth of invasion, (C) pure undifferentiated type, (D) mixed type, (E) lymphovascular invasion, and (F) ulceration as risk factors for LNM.

Tumor size, depth of invasion, histological type, and ulceration are assessed as criteria for ESD treatment of EGC [10]; thus, we performed multivariate analysis using these 4 covariates to determine their effect on the rate of LNM. The multivariate analyses conducted in 4 studies revealed the risk factors for LNM in EGC patients, the results of which were pooled. Fig 5 presents the forest plots generated for each individual risk factor. The multivariate analyses show that tumor size (>2 cm vs. ≤2 cm: OR = 2.05, 95% CI: 1.06–3.94, z = 2.14, p = 0.032; I² = 83.2%, p = 0.003, random-effect; Fig 5A), depth of invasion (SM vs. M: OR = 3.00, 95% CI: 2.16–4.16, z = 6.58, p = 0.000; I² = 22.9%, p = 0.273, fixed-effect; Fig 5B), PU (PU vs. PD: OR = 1.89, 95% CI: 1.49–2.40, z = 5.21, p = 0.000; I² = 28.4%, p = 0.232, fixed-effect; Fig 5C), mixed type (mixed vs. PD: OR = 2.96, 95% CI: 2.24–3.92, z = 7.58, p = 0.000; I² = 0%, p = 0.836, fixed-effect; Fig 5D), lymphovascular invasion (present vs. absent: OR = 7.68, 95% CI: 6.17–9.56, z = 18.27, p = 0.000; I² = 0%, p = 0.512, fixed-effect; Fig 5E), and ulceration (present vs. absent: OR = 1.82, 95% CI: 1.42–2.32, z = 4.78, p = 0.000; I² = 47.5%, p = 0.168, fixed-effect; Fig 5F) were independent risk factors for LNM in EGC patients, with no heterogeneity. Among the identified independent risk factors, lymphovascular invasion was found to be most strongly related to LNM (OR = 7.68, 95% CI: 6.17–9.56). Furthermore, the mixed type (OR = 2.96, 95% CI: 2.24–3.92) was more related to LNM than PU (OR = 1.89, 95% CI: 1.49–2.40). Fig 5F shows that the presence of ulcers was also a dependent risk factor for LNM; however, Zhong et al. [58] found that ulcers were not a contributing factor (p = 0.256).

## 4. Discussion

The presence of LNM determines the feasibility of endoscopic resection in patients with EGC and substantially harms their prognosis, and the rate of LNM is a key factor in deciding the treatment course for EGC [65–67]. In a multicenter study in Japan [68], lesions meeting the indications for ESD had an LNM rate of 0% and favorable long-term outcomes. We conducted this meta-analysis to calculate the LNM rate in mixed-type EGC patients, and the findings suggest that these patients may not be suitable for ESD. The Japanese guidelines divide the endoscopic resection into a differentiated histological type and an undifferentiated histological type; therefore, we analyzed the studies in terms of MD and MU.

The overall rate of LNM for MD lesions was 12%. The limited availability of data from the studies of mucosal EGC prevented meta-analysis, though the rate was mentioned by several articles. Takizawa et al. [15] suggested an LNM rate of 3.1% in PD and 11.1% in MD (p = 0.066), and Lee et al. [49] presented rates of 0.5% in PD and 5.1% in MD (p<0.001), proving MD to be strongly associated with LNM. The incidence of LNM in submucosal EGC patients was considerably higher than in mucosal EGC patients. The pooled rate of LNM in MD was 20%, and the rates in PU and PD were 21% and 13%, respectively. Interestingly, the rate in MD was much higher than in PD and nearly the same as PU. The Japanese guidelines [10] classify MD as differentiated to follow the indications. Furthermore, curative ESD resection is thought to present a very low risk of LNM in EGC. However, our results show that the indications for PD do not seem suitable for MD. Zhong et al. [58] concluded that for mucosal EGC, the mixed-type group and PU group showed similar clinicopathologic features and aggressive behavior of mucosal EGC and could therefore be managed in the same way. However, the data were lacking, so we could not find any further details. For submucosal EGC, the Japanese guidelines state that MD in SM1 meets the curative resection criteria (eCura B) after ESD. In contrast, our study found that the depth of submucosal invasion (OR 3.0) and mixed-type EGC (OR 2.96) are independent risk factors for LNM, and when both factors occur simultaneously, the rate of LNM is elevated. Horiuchi [50] also proved MD to be a risk factor for non-curative resection, irrespective of lesion size, and Shim [57] calculated the curative resection rate for MD in SM1 as only 60%. These results are not compatible with the Japanese guidelines. In submucosal EGC, MD histology may be a separate prognostic consideration after endoscopic resection and the indications of MD for ESD should be stricter than where they currently stand.

Similarly, the MU type also had a high prevalence of LNM (22%). Takizawa [15] showed that in mucosal EGC, LNM was significantly more common in MU-type (19.0%) than in PU-type (6.1%) lesions (p = 0.006). Horiuchi et al. [54] indicated that LNM did not occur for MU tumors with a diameter of 1–30 mm in mucosal gastric cancer; this was confirmed by Zhong [58], who stated that MU could be treated as PD. Endoscopic therapy is not considered for submucosal EGC with undifferentiated histology according to the Japanese guidelines. However, our pooled LNM rate in MU was 33%, much higher than in PU (21%); thus, submucosal MU EGC represents non-curative ESD (eCura C).

Our study has several limitations. First, there are insufficient statistics for mixed-type mucosal EGC. Based on the studies mentioned above, the mixed types (MD and MU) should be further considered for ESD treatment in mucosa, though whether mixed-type mucosal EGC can be embedded in the indication for endoscopic resection remains unclear. Second, to determine more detailed indications of MD in submucosal EGC, the depth of invasion should be considered, and LNM in T1b1 and T1b2 should be analyzed separately. However, subdividing the depth of invasion is difficult, and several statistics still should be studied regarding the mixed type in T1b1 and T1b2 EGC, which will help evaluate the treatment effect of mixed-type submucosal gastric cancer.

## 5. Conclusion

Our meta-analysis demonstrated that mixed-histological EGC has a higher rate of LNM than the pure types. We further highlighted the high rate of LNM in MD EGC, which suggests that it is unreasonable for MD EGC to be treated as PD EGC in the indications for ESD.

## Supporting information

S1 Fig (TIF)

S2 Fig (TIF)
