# Supplementary figures and images for: Relationship between histological mixed-type early gastric cancer and lymph node metastasis: A systematic review and meta-analysis
Source: PLoS One. 2022 Apr 15;17(4):e0266952. doi: 10.1371/journal.pone.0266952 (PMC9012370; doi:10.1371/journal.pone.0266952)

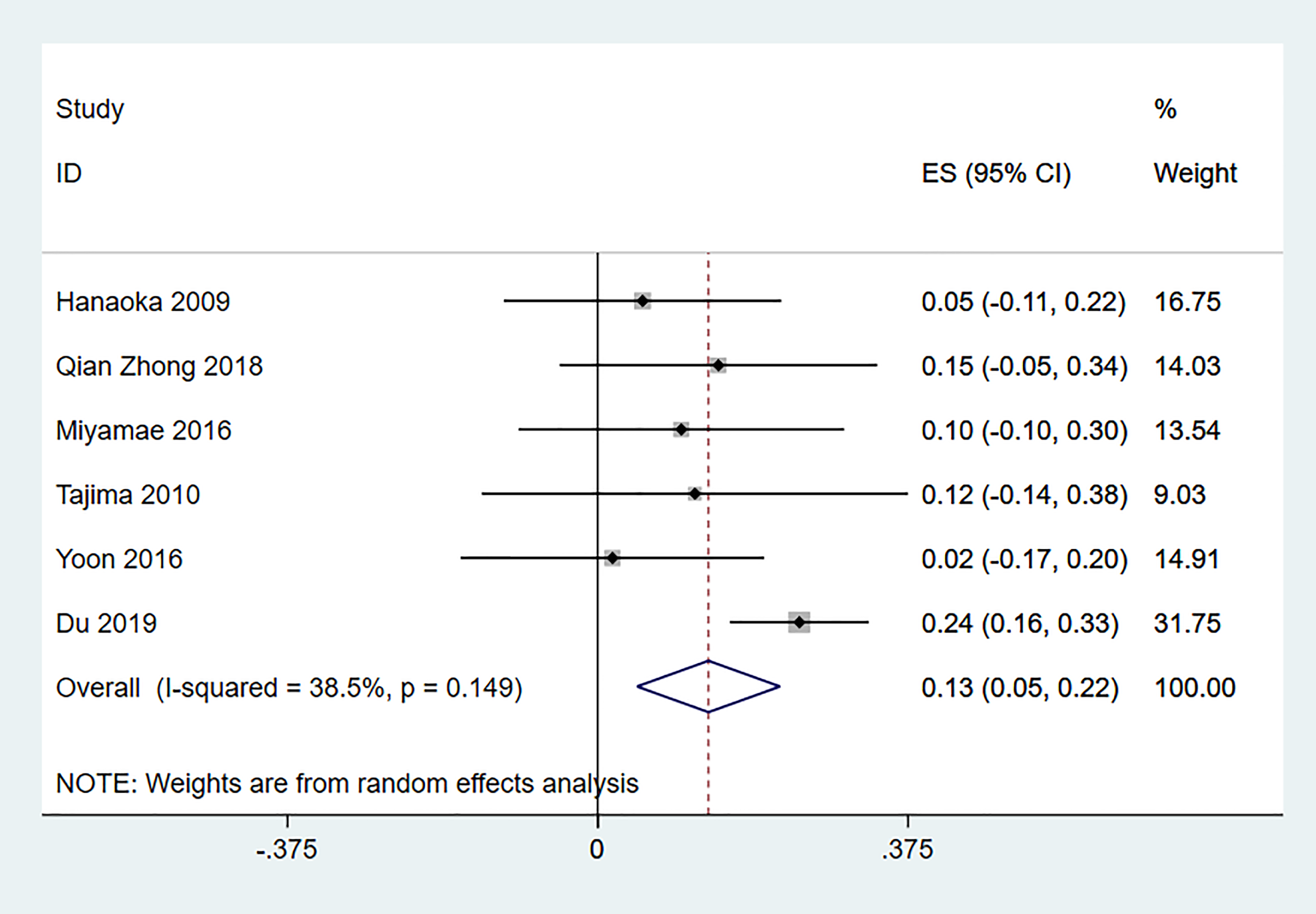

Supplement: S1 Fig — (TIF) [file pone.0266952.s001.tif]

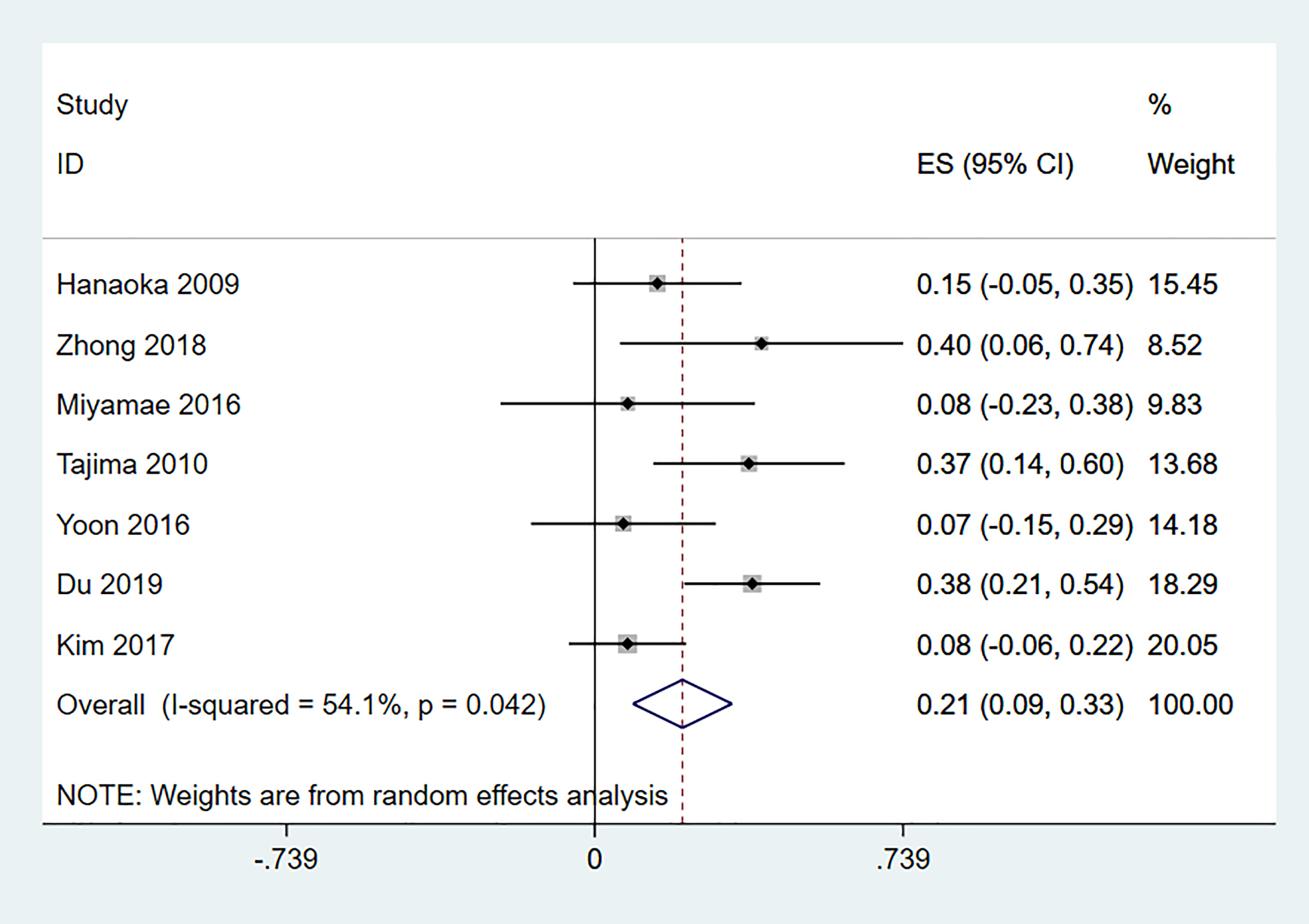

Supplement: S2 Fig — (TIF) [file pone.0266952.s002.tif]
